# Intracranial Bleeding as an Atypical Clinical Presentation of Choledochal Cyst in a Young Infant

**DOI:** 10.1097/PG9.0000000000000021

**Published:** 2020-11-11

**Authors:** Farah F. Kadhim Al Khawaja, Mohammed H. A. Nasser Al-Amri, Fatihi Hassan Soliman Toaimah

**Affiliations:** From the * Division of Pediatric Emergency Medicine, Department of Pediatrics, Hamad Medical Corporation, Doha, Qatar; † Department of Clinical Pediatrics and Clinical Emergency Medicine, Weill Cornell Medical College in Qatar, Cornell University, Doha, Qatar.

## INTRODUCTION

Choledochal (biliary) cysts are congenital anomalies involving extrahepatic and/or intrahepatic biliary system (1). Some may be asymptomatic but many others can cause significant morbidity and mortality unless discovered and treated early. The occurrence is higher in Asian nations, with predominance in females, and about two-thirds of the patients are seen in Japan (1). They are considered as benign cysts however their diverse clinical presentations may pose a challenge to the early diagnosis and treatment.

## CASE REPORT

A previously healthy 83-day-old Indian male infant, presented to the emergency department with abnormal lip movements, uprolling of the eyes and tonic-clonic convulsions. His seizures lasted more than 35 minutes, for which he received 2 doses of benzodiazepines, phenytoin and phenobarbitone, and was connected to mechanical ventilation.

His vital signs were heart rate 158 bpm, respiratory rate 48 bpm, temperature 37.2°C, blood pressure 82/42 mmHg, and oxygen saturation above 95%. He was very pale, not jaundiced, has no dysmorphic features, reactive pupils bilaterally, full tense anterior fontanel with a bruise on the left frontal area, and no palpable abdominal mass. Otherwise, systemic examination was not significant. There was history of light-colored stool and urine became dark. The birth weight was 2.5 kg and the current weight was 4.65 kg denoting that he has failure to thrive as the weight for age is below −2SD. The provisional diagnosis initially was suspected child abuse, sepsis, or genetic/metabolic defect.

Initial blood investigations revealed hemoglobin level 5.1 g/dL, normal white blood cell and platelets counts. Coagulation tests were prolonged: PT >120 seconds (normal up to 14.3), INR >10 (high > 4.9), PTT >180 seconds (normal up to 55). Liver function tests: total bilirubin 44 µmol/L (normal 0–21 µmol/L), direct serum bilirubin 32 µmol/L (normal 0–7 µmol/L), alanine aminotransferase, and aspartate aminotransferase were normal, and alkaline phosphatase 1481 µ/L (up to 1000). CRP was <5 mg/L, respiratory viral panel was negative, and blood culture showed no growth.

**FIGURE 1.**
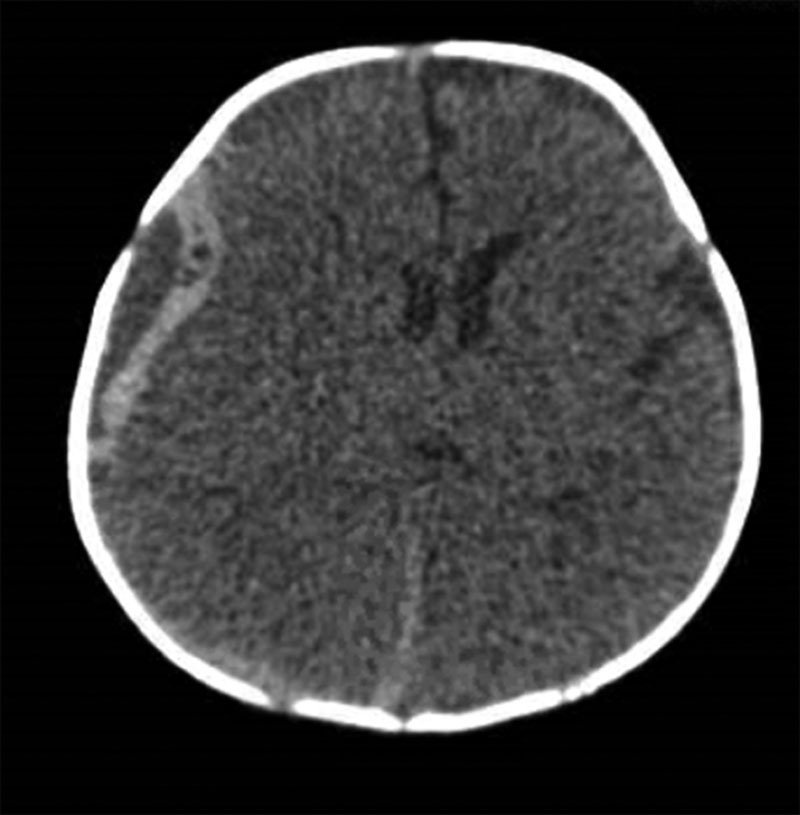
A non-contrast cranial CT showing hyperdense biconvex collection suggesting hematoma with midline displacement.

**FIGURE 2.**
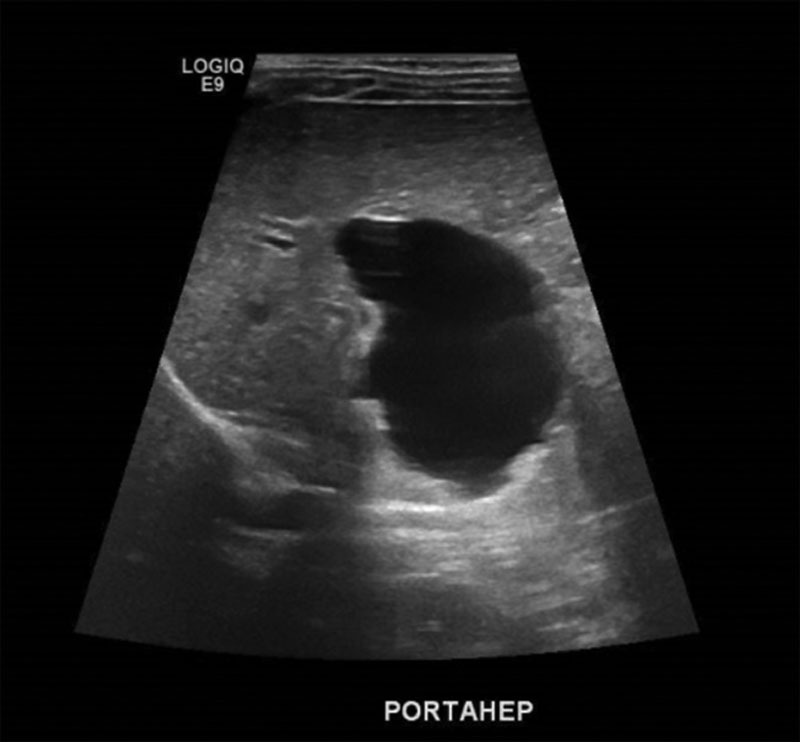
Ultrasonography of the abdomen showing well defined cystic area at the porta hepatis.

Head CT scan showed mixed density extra-axial subdural hematoma along the right cerebral convexity measuring 10 mm in maximum thickness (Fig. 1). Abdominal ultrasound showed biliary ductal dilation along with dilated common bile duct (CBD) of 7 mm, connected to an oval irregular 4 × 3 cm cystic structure in the right hypochondrium (Fig. 2).

The infant received 3 units of packed red blood cells, 3 doses of intravenous vitamin K, and 2 units of fresh frozen plasma. His hemoglobin level and coagulopathy were corrected shortly.

Approximately 6 days after admission, the blood investigations showed a rise in aspartate aminotransferase up to 110 µ/L, total bilirubin up to 196 µmol/L, and direct bilirubin up to 180 µmol/L. As a result, the patient started to have jaundice. The patient developed low-grade fever and received a course of intravenous antibiotics for a clinically suspected sepsis, which may explain the increase in liver enzymes. Gamma-glutamyl transferase was 2540 µ/L (normal up to 122 µ/L) on the eighth day of admission.

**FIGURE 3.**
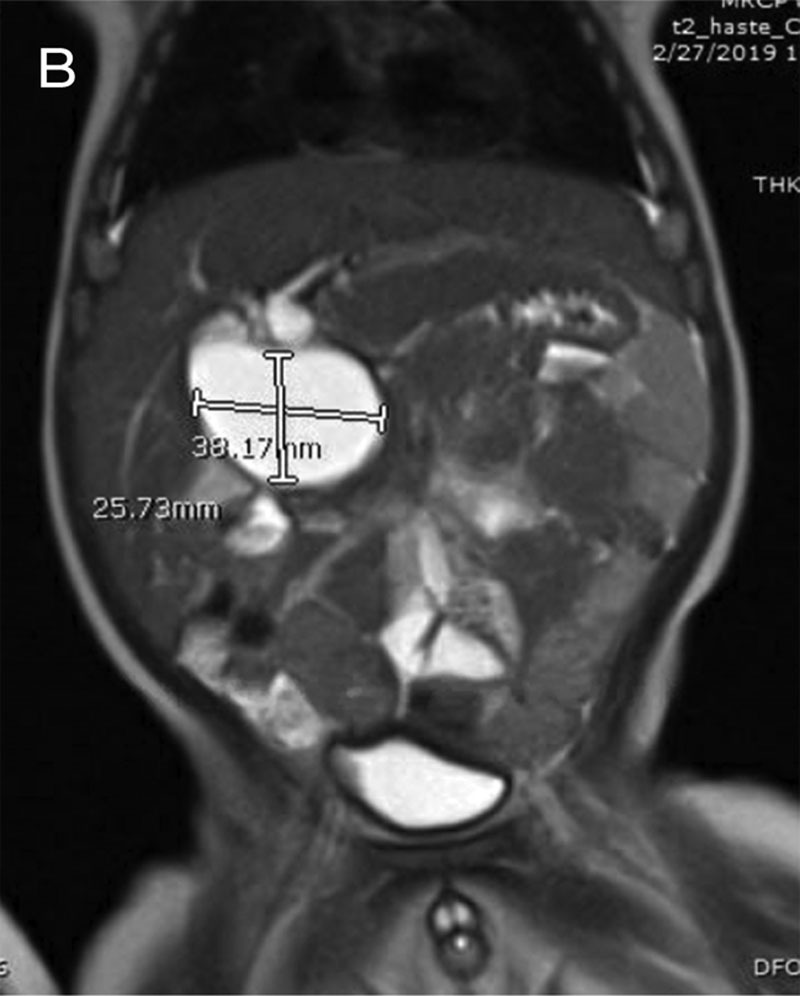
MRCP coronal image of the choledochal cyst. MRCP, magnetic resonance cholangiopancreatography.

Magnetic resonance cholangiopancreatography showed saccular-cystic dilatation of the CBD measuring 4.2 × 2.5 × 3 cm, extending to its terminal part. The common hepatic duct, the part of CBD proximal to the cystic dilatation, also appear dilated, measuring about 8 mm (Fig. 3). Features were suggestive of choledochal cyst type I in agreement with Todani’s classification.

Laparoscopic cyst removal was failed as the CO_2_ had climbed with acidosis, and was converted to open laparotomy. Complete surgical excision of the cyst was successfully done followed by hepaticoduodenostomy. The laparotomy revealed a cirrhotic liver with minimal ascites in the peritoneal cavity.

Intraoperative liver biopsy was performed and revealed marked cholangiolar proliferation and bile plug progressing to biliary cirrhosis. Sections showed a duct with a larger caliber than the gallbladder with focal bile staining and some denudation of the epithelium.

The baby did not require craniotomy. Cranial CT scan after 3 months showed no intracranial hemorrhage (ICH), and abdominal ultrasound after 10 months showed no biliary dilatation. The patient was not on anticonvulsants at 16 months postoperative, has no residual neurological deficits, and his blood investigations were normal for age.

## DISCUSSION

Choledochal cysts are congenital anomalies of the biliary tree, classified into 6 main types with some recognized subtypes, of which type I is the most common and accounts for 50–80% (1,2). They may be associated with other congenital abnormalities in the biliary tree or elsewhere in other body organs like the heart, gastrointestinal tract, kidneys, or other developmental defects (3).

The classic set of the triad of a palpable mass, jaundice and abdominal pain is no longer the most common presenting features as the majority could present with only 1 or 2 of these (4). Others may present with nonspecific symptoms as nausea, vomiting, fever, pruritus, and weight loss or may have their first symptoms related to the complications such as bleeding tendency (5). Even coincidental finding while doing investigations for other reasons has been reported (6).

Abdominal ultrasound is the first imaging study that can identify the cyst, which is usually confirmed by more detailed radiology like magnetic resonance imaging, or magnetic resonance cholangiopancreatography which is regarded as the best because it is noninvasive, has no risk of ionizing radiation, and gives good details about the biliary tree and the type of the defect and it is usually done before surgery (7).

Complete surgical excision of the cyst, if possible, is the best modality of treatment. This will improve the outcome and decrease the risk of the long life complications especially malignancy (2).

This case represents a rare and complicated presentation of choledochal cyst in which the coagulopathy is attributed to malabsorption of vitamin K secondary to cholestasis, liver dysfunction, and cirrhosis. Late vitamin K deficiency, between 2 and 12 weeks of age, has been reported with biliary atresia but very rarely with choledochal cysts (8). This bleeding is usually rapidly corrected by the administration of vitamin K. To our knowledge, only 3 cases of ICH secondary to choledochal cyst has been described in the literature and all presented initially with jaundice (5,9,10). This case presented with convulsions without jaundice which has made the hepatobiliary involvement as a cause of coagulopathy and ICH a remote initial possibility of choledochal cyst.

## CONCLUSION

A young infant presented ICH as the earliest symptom of choledochal cyst. He suffered from coagulopathy secondary to liver dysfunction which resolved with early vitamin k administration. This case highlights the importance of recognition and maintaining a high index of suspicion in preicteric liver disease presented with ICH as the initial clinical manifestation.

## Acknowledgments

The authors would like to thank Dr. Vishwanatha Kini for his help in preparing the radiological images.

## References

[1] Singham J Yoshida EM Scudamore CH. Choledochal cysts: part 1 of 3: classification and pathogenesis. Can J Surg. 2009; 52:434–440 19865581 PMC2769090

[2] Jabłońska B. Biliary cysts: etiology, diagnosis and management. WJG. 2012; 18:4801 23002354 10.3748/wjg.v18.i35.4801 PMC3447264

[3] Gupta L Bhatnagar V. A study of associated congenital anomalies with biliary atresia. J Indian Assoc Pediatr Surg. 2016; 21:10–13 26862288 10.4103/0971-9261.158095 PMC4721121

[4] Lipsett PA Pitt HA Colombani PM. Choledochal cyst disease. A changing pattern of presentation. Ann Surg. 1994; 220:644–652 7979612 10.1097/00000658-199411000-00007 PMC1234452

[5] Fumino S Iwai N Deguchi E. Bleeding tendency as a first symptom in children with congenital biliary dilatation. Eur J Pediatr Surg. 2007; 17:2–5 17407013 10.1055/s-2007-964928

[6] Yadav AK Zhan J Zhang S. Congenital solitary intrahepatic biliary cyst in infant: a rare case report. J Pediatr Surg Case Rep. 2018; 30:64–67

[7] Özkan Gezer H. Shehata S. Pediatric choledochal cysts: unknowns are decreasing. In: Pediatric Surgery, Flowcharts and Clinical. Algorithms. 2019. IntechOpen

[8] Akiyama H Okamura Y Nagashima T. Intracranial hemorrhage and vitamin K deficiency associated with biliary atresia: summary of 15 cases and review of the literature. Pediatr Neurosurg. 2006; 42:362–367 17047416 10.1159/000095566

[9] Waqas Ali S Manzoor N Ashraf MS. Intra-cerebral hemorrhage associated with choledochal cyst in an infant. J Pediatr Surg Case Rep. 2020; 58:101373

[10] Chen TY Wang HK Yeh ML. Subdural hemorrhage as a first symptom in an infant with a choledochal cyst: case report. J Neurosurg Pediatr. 2012; 9:414–416 22462707 10.3171/2011.12.PEDS11279

